# Supplementary material for: Ovary Dissection Is a Sensitive Measure of Sterility in Anopheles gambiae Exposed to the Insect Growth Regulator Pyriproxyfen
Source: Insects. 2023 Jun 14;14(6):552. doi: 10.3390/insects14060552 (PMC10299475; doi:10.3390/insects14060552)
Supplement: Supplementary file 1 [file insects-14-00552-s001.zip › insects-2432266-supplementary.pdf]

Table S1. Sterile and fecund *An. gambiae* RSP females by method and treatment group with the sensitivity, specificity, positive predictive value (PPV), and negative predictive value (NPV).

	**Oviposition**			**Dissection**		
	**PPF**	**Untreated**	**N total (%)**	**PPF**	**Untreated**	**N total (%)**
**N sterile (%)**	113 (40.6)	133 (47.8)	246 (88.5)	54 (40.3)	38 (28.4)	92 (68.7)
**N fecund (%)**	1 (0.4)	31 (11.2)	32 (11.5)	0 (0.0)	42 (31.3)	42 (31.3)
**N total (%)**	114 (41.0)	164 (59.0)	278 (100.0)	54 (40.3)	80 (59.7)	134 (100.0)
**Sensitivity (%)**	99.1			100.0		
**Specificity (%)**	18.9			52.5		
**PPV (%)**	45.9			58.7		
**NPV (%)**	96.9			100.0		

Table S2. Prediction of PPF exposure to *An. gambiae* females during blinded dissections.

	**Cylinder Assays**				**Tunnel Tests**				
**Prediction**	**Untreated**	**PPF**	**Olyset Net**	**Olyset Duo 20W**	**Untreated**	**PPF**	**PPF 20W**	**Olyset Net**	**Olyset Duo 20W**
N true prediction	105	100	117	14	68	82	23	23	23
N false prediction	7	3	8	60	6	9	0	1	0
N with mixed eggs (no prediction)	3	2	2	10	1	3	0	0	4
Unpredicted (%)	2.6	1.9	1.6	11.9	1.3	3.2	0.0	0.0	14.8
**Correct prediction (%)**	93.8	97.1	93.6	18.9	91.9	90.1	100.0	95.8	100.0

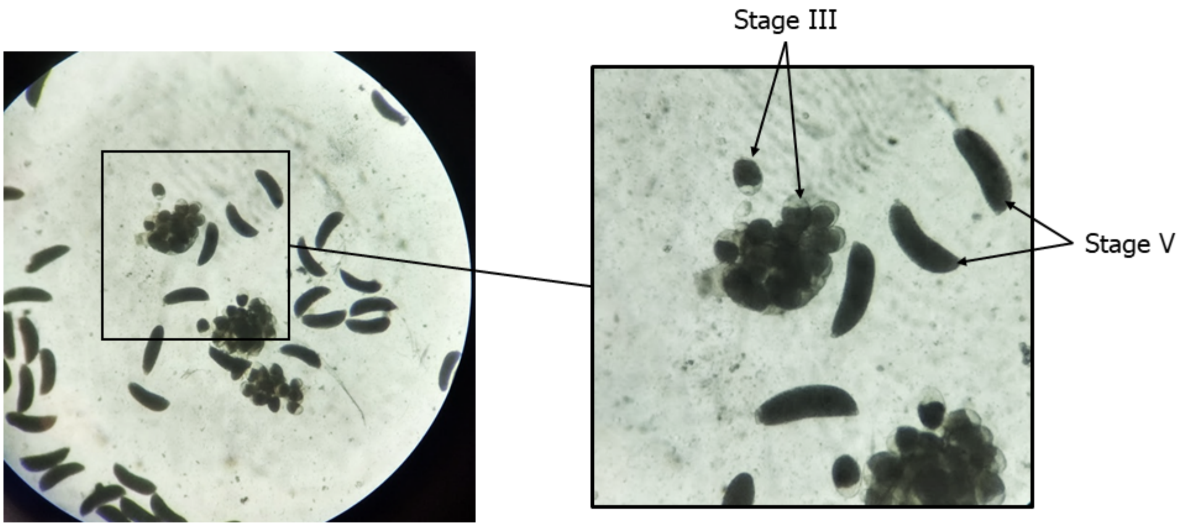

Figure S1. Image taken from stereoscopic microscope comparing dissected eggs at Christophers' stage III (underdeveloped) and stage V (developed).
